# A three-dimensional in vitro model of erythropoiesis recapitulates erythroid failure in myelodysplastic syndromes

**DOI:** 10.1038/s41375-019-0532-7

**Published:** 2019-08-02

**Authors:** Edda María Elvarsdóttir, Teresa Mortera-Blanco, Marios Dimitriou, Thibault Bouderlique, Monika Jansson, Isabel Juliana F. Hofman, Simona Conte, Mohsen Karimi, Birgitta Sander, Iyadh Douagi, Petter S. Woll, Eva Hellström-Lindberg

**Affiliations:** 1 Center for Hematology and Regenerative Medicine, Karolinska Institutet, Department of Medicine, Karolinska University Hospital Huddinge, Stockholm, Sweden; 2 Division of Pathology, Department of Laboratory Medicine, Karolinska Institutet, Karolinska University Hospital Huddinge, Stockholm, Sweden

**Keywords:** Regenerative medicine, Anaemia

## Abstract

Established cell culture systems have failed to accurately recapitulate key features of terminal erythroid maturation, hampering our ability to in vitro model and treat diseases with impaired erythropoiesis such as myelodysplastic syndromes with ring sideroblasts (MDS-RS). We developed an efficient and robust three-dimensional (3D) scaffold culture model supporting terminal erythroid differentiation from both mononuclear (MNC) or CD34^+^-enriched primary bone marrow cells from healthy donors and MDS-RS patients. While CD34^+^ cells did not proliferate beyond two weeks in 2D suspension cultures, the 3D scaffolds supported CD34^+^ and MNC erythroid proliferation over four weeks demonstrating the importance of the 3D environment. CD34^+^ cells cultured in 3D facilitated the highest expansion and maturation of erythroid cells, including generation of erythroblastic islands and enucleated erythrocytes, while MNCs supported multi-lineage hemopoietic differentiation and cytokine secretion relevant for MDS-RS. Importantly, MDS-RS 3D-cultures supported de novo generation of ring sideroblasts and maintenance of the mutated clone. The 3D cultures effectively model a clonal disease characterized by terminal erythroid failure and can be used to assess therapeutic compounds.

## Introduction

Terminal erythroid differentiation involves a series of morphologically distinct maturation stages of erythroblasts that following terminal enucleation are released as reticulocytes from the bone marrow (BM) into the blood stream where they mature into erythrocytes [1, 2]. The formation of erythroblastic islands, i.e., erythroblasts surrounding a macrophage that phagocytoses the extruded nuclei while providing nutrients and iron for heme synthesis, is critical for this process [3–5]. It has been a challenge to establish in vitro models that fully recapitulate terminal erythropoiesis, limiting the elucidation of regulatory mechanisms involved during healthy and defective erythropoiesis, an important prerequisite for identifying novel therapy targets for patients with anemia.

Myelodysplastic syndromes (MDS) are hematopoietic malignancies frequently leading to severe anemia and chronic dependency on red blood cell transfusions [6]. We have focused our research on the subgroup MDS with ring sideroblasts (MDS-RS), characterized by ineffective terminal erythroid differentiation, recurrent somatic mutations in splicing factor 3b subunit 1 (*SF3B1*) [7–9] and accumulation of aberrant mitochondrial ferritin in erythroblasts, resulting in the generation of ring sideroblasts (RS) constituting 15–50% of nucleated erythroid bone marrow (BM) cells [10–14]. Using suspension cultures, we have recapitulated healthy and MDS-RS erythropoiesis up to the stage of intermediate erythroblasts, with increased mitochondrial ferritin and mitochondria-mediated apoptosis [11, 15, 16]. However, to further elucidate biological consequences of *SF3B1* mutations for terminal erythropoiesis would require efficient in vitro generation of RS and enucleated erythrocytes from MDS-RS stem and progenitor cells.

Our group has previously generated human RS following xeno-transplantation of purified hematopoietic stem cells (HSC) into immune-compromised mice, demonstrating that human HSCs are the initiating cells of the MDS-RS phenotype and able to differentiate within the mouse BM [17]. In addition, formation of human RS was recently reported in an MDS patient-derived xenotransplantation model using cytokine-humanized immunodeficient mice lacking the corresponding murine cytokines [18]. While the xenograft models could be a valuable in vivo platform for studying dysregulated erythroid development in MDS-RS, low engraftment and recovery of MDS-RS erythroid cells from the mice limit possible experimental outputs [19, 20]. Furthermore, genetic mouse models allowing conditional expression of mutant SF3B1 have failed to generate RS, reflecting that downstream effects of mutations may differ between mice and man [21, 22].

Based on the above-described limitations we set out to develop an in vitro three-dimensional (3D) system allowing for longitudinal studies of maturing erythroid cells, particularly focused on the formation of erythroblastic islands and enucleated erythrocytes. As the goal was to study human disease with erythroid failure we cultured primary cells from healthy individuals (normal bone marrow; NBM) and patients with MDS-RS, a disease with an easily quantifiable phenotype. Modeling the whole process of MDS-RS erythropoiesis requires, in addition to RS formation, maintenance of the *SF3B1* mutated clone throughout differentiation, similar to what we demonstrated in freshly aspirated BM from MDS-RS patients [11]. For this purpose we modified a previously described system using collagen coated polyurethane scaffolds with a pore size and distribution of similar architecture to human BM for erythroid growth and differentiation [23, 24]. We compared seeding freshly isolated CD34^+^ enriched cells, a source that has been used to assess early and intermediate erythroid differentiation in liquid cultures [15, 16, 25–27], to freshly separated mononuclear cells (MNCs) that contain cells of multiple hematopoietic lineages, including mesenchymal stromal cells. In order to assess the effect of the 3D scaffold system per se, we cultured the same cells in suspension (2D) with the same medium. To facilitate erythropoiesis we based the medium composition on two weeks of erythroblast media, previously used to facilitate growth of erythroid progenitors in liquid culture [15, 16, 26], followed by two weeks of simplified medium with iron supplementation to facilitate erythroid maturation [28, 29].

We demonstrated that while primary CD34^+^ cells could only be maintained for two weeks in suspension culture, 3D scaffolds allowed them to continuously expand and undergo complete differentiation and enucleation over a four week culture period. While the 3D CD34^+^ culture mainly facilitated erythroid maturation, the corresponding MNC cultures supported a more variable hematopoietic differentiation, both for healthy and MDS-RS samples. All 3D cultures supported formation of erythroblastic islands and enucleation and importantly MDS-RS erythropoiesis with maintained *SF3B1* mutated allele burden and development of key dysplastic features of terminal maturation. Our results show that the 3D scaffold system can be used to model erythroid differentiation from healthy and MDS progenitors, and indicates its usefulness for other disorders with defective erythroid maturation and for assessment of therapeutic compounds aiming to improve erythroid output.

## Materials and methods

### Sample collection and culture conditions

BM aspirates were obtained from eight healthy donors (NBM) and eleven patients with MDS-RS, according to the World Health Organization 2016 revision of the classification of myeloid neoplasms and acute leukemia [10]. Patients were diagnosed using a multi-professional conference approach. Healthy controls and patients sampled at the Hematology Department at Karolinska University Hospital, Sweden, provided informed consent and the study was approved by the Ethics Research Committee at Karolinska Institutet (2010/427-31/1 and 2011/1257-31/1). All MDS-RS patients were *SF3B1* mutated and had non-transfusion dependent anemia (Supplementary Table 1).

**Fig. 1:**
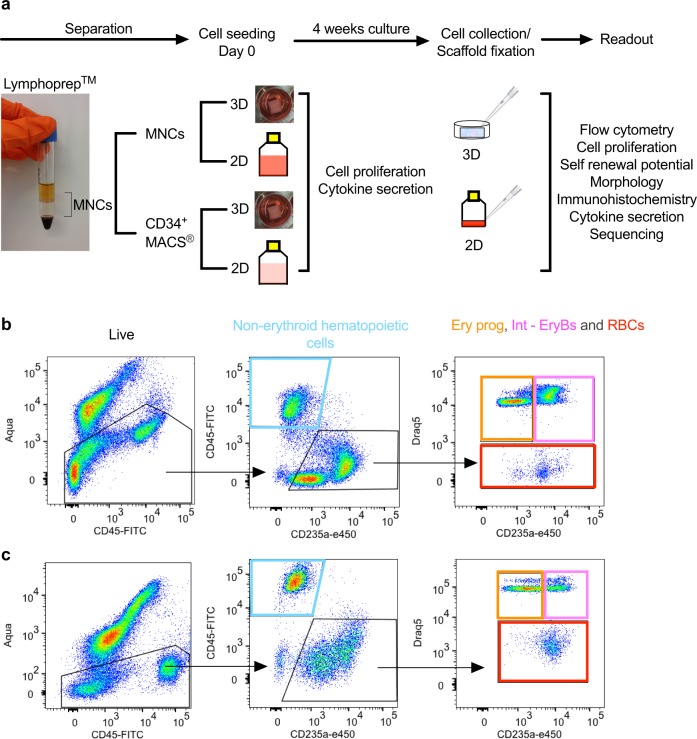
Culture setup and flow cytometry strategy. **a** Scheme illustrating separation of the mononuclear cell (MNC) fraction, enrichment of CD34^+^ cells and seeding into scaffolds (3D) or suspension (2D) cultures, followed by 4 weeks of culture after which cells were aspirated and experiments performed. **b** Representative flow cytometry analysis for quantifying non-erythroid hematopoietic cells, erythroid progenitor cells (Ery prog), intermediate erythroblasts (Int-EryBs) and enucleated erythrocytes (RBCs) after 4 weeks of 3D CD34^+^ NBM culture. **c** Representative flow cytometry analysis for quantifying non-erythroid hematopoietic cells, erythroid progenitor cells (Ery prog), intermediate erythroblasts (Int-EryBs) and enucleated erythrocytes (RBCs) after 4 weeks of 3D CD34^+^ MDS-RS culture (see Supplementary Fig. 3 for representation of the other culture conditions)

BM MNCs were isolated using Lymphoprep (Nycomed, Oslo, Norway) density gradient centrifugation. To assess the effect of a richer cellular variety both the layered MNCs and the Lymphoprep fraction were harvested (Fig. 1a). A fraction of the MNCs were enriched for CD34^+^ cells using magnetic-activated cell sorting (MACS; Miltenyi Biotec, Bergish Gladbach, Germany) according to manufacturer’s protocol, resulting in CD34^+^ purity higher or equal to 75%. MNCs were seeded either directly into 24-well tissue culture plates (2D MNC) or into sterile scaffolds (3D MNC) placed in 24-tissue culture plates at a concentration of 2 × 10^6^ cells/0.1 ml medium and incubated for 15 minutes at 37 °C and 5% CO_2_ before adding 1.4 ml of medium into each well. In order to compare the growth of BM MNCs vs. CD34^+^ in the 3D system we ensured that an equivalent number of CD34^+^ cells were seeded in both types of scaffolds. For each sample we calculated the percentage of CD34^+^ cells in the MNCs used for enrichment and seeded a number of CD34^+^ cells corresponding to 2 × 10^6^ MNCs into each CD34^+^ scaffold (Supplementary Table 2). Cells in erythroblast suspension culture (2D CD34^+^) were seeded at a concentration of 0.1 × 10^6^ cells/ml media as previously well-described [15, 16]. A description of medium composition and exchange can be found in Supplementary Methods.

### Scaffold fabrication and sterilization

Scaffolds made of polyurethane were fabricated using thermally induced phase separation of polymer solutions (5% weight/volume) followed by solvent sublimation as previously described [23, 30]. For further details see Supplementary Fig. 1 and Supplementary Methods.

### Flow cytometry

Non-erythroid hematopoietic cell, erythroid progenitor cell, intermediate erythroblast and enucleated erythrocyte analysis was performed as described in Supplementary Methods and in Supplementary Table 3.

### Cell proliferation

To assess changes in number of metabolically active cells in the cultures we used a tetrazolium compound [3-(4,5-dimethylthiazol-2-yl)-5-(3-carboxymethoxyphenyl)-2-(4-sulfophenyl)-2H-tetrazolium], CellTiter 96® AQueous One Solution Proliferation Assay, (MTS, Promega, Madison, WI, USA) at the day of seeding and at the end of weeks 2, 3, and 4 as previously described [24]. For further details see Supplementary Methods.

### Functional stem and progenitor cell assays

Detailed methods for long-term culture colony-forming cell (LTC-CFC) assays are described in Supplementary Methods.

### Pyrosequencing

Pyrosequencing (PyroMark Q24 system; Qiagen, Hilden, Germany) was used to confirm and quantify allele burden of heterozygous *SF3B1* single nucleotide mutations that were identified by targeted sequencing methodologies [17, 31]. Sequencing primers have been previously validated [11] and sequence information is provided in Supplementary Table 4. For further details see Supplementary Methods.

### Morphological and histopathological evaluation

Seeded cells from day 0 and extracted from week 2 and 4 of cultures were either stained with May-Grünwald Giemsa or Perl´s Prussian blue using standard pathology procedures. An MDS pathologist counted RS blindly, without information about the culture system, and calculated their percentage of total nucleated erythroblasts.

Scaffolds after 4 weeks of culture were fixed in 4% paraformaldehyde overnight and either embedded in paraffin, as previously described [24], or cryoprotected with 30% sucrose overnight and embedded in Tissue-Tek^®^ O.C.T. Compound (Sakura Finetek Europe B.V., AJ Alphen aan den Rijn, The Netherlands) and frozen with dry ice before storage at −80 °C. Cells stained with May-Grünwald Giemsa, Perl´s Prussian blue and H&E stained scaffold sections were scanned using the Panoramic MIDI II scanner and Panoramic Viewer software (3D Histech, Budapest, Hungary). Images were taken using an AxioCam MRm (Carl Zeiss, Oberkochen, Germany) at ×40 magnification. Images of fluorescently stained scaffold sections were acquired using a NIKON (Nikon Instruments Europe BV, Amsterdam, The Netherlands) single-point scanning confocal equipped with GaAsp detectors, spectral detector, ×10, ×20, ×40, and ×63 objectives. Sections were imaged immediately after staining to avoid fluorophore dimming. Images were acquired in the .nd2 format and processed with the NIS Element software from NIKON systems. Staining details can be found in Supplementary Methods.

### Cytokine measurements

TGF-β1, GDF11, IL-10, and IL-1α were measured in medium collected from week 1, 2, and 4 of three NBM and three MDS-RS MNC and CD34^+^ 3D cultures. A detailed description of methods used for detection can be found in Supplementary Methods.

### Statistical analysis

All results are plotted as means ± SEM and statistical analysis was performed using the GraphPad Prism 6 software. One-way analysis of variance (ANOVA) followed by Tukey’s honest significant difference (HSD) test for multiple comparisons was used to compare three or more datasets, two-way ANOVA followed by Sidak´s multiple comparisons test was used to compare two datasets and unpaired two-tailed *T*-tests were used to compare datasets if one group included values below detection levels only. *P* values < 0.05 were considered significant. *p < 0.05, **p < 0.01, and ***p < 0.001.

## Results

### Establishment of erythroid cultures and cell composition

**Fig. 2:**
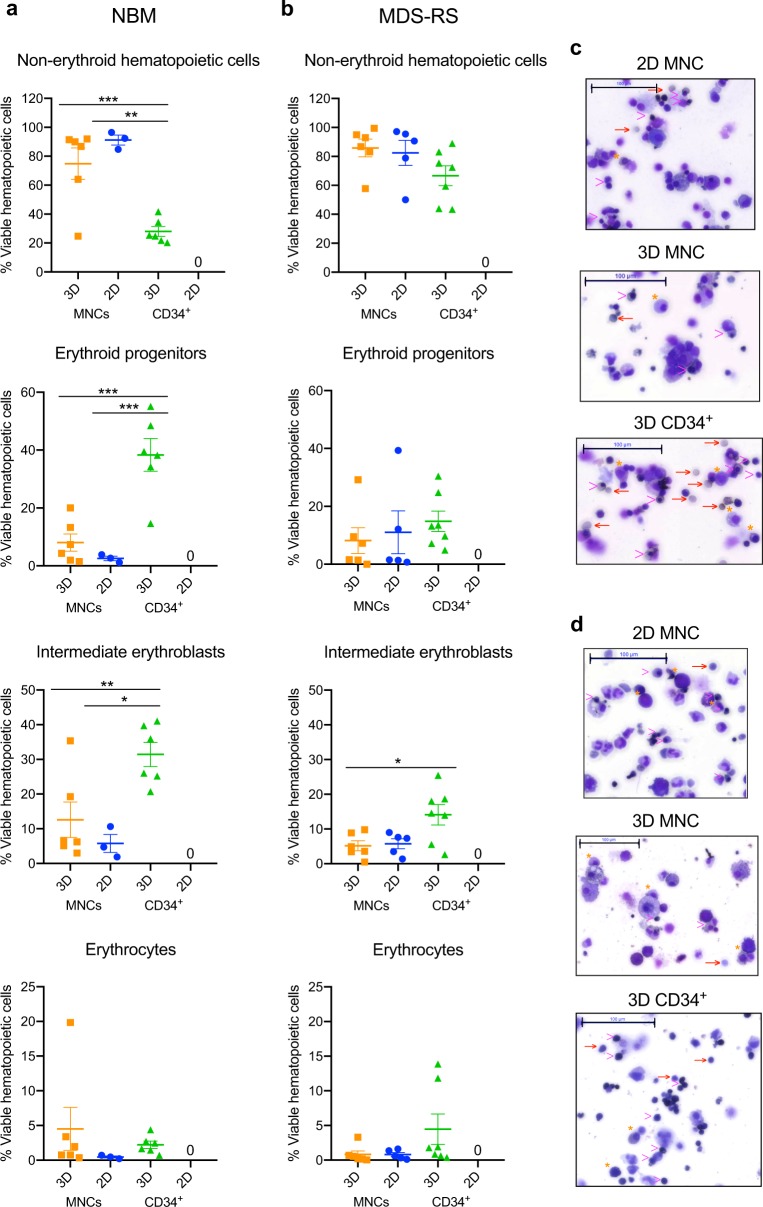
Cell composition after four weeks of NBM and MDS-RS cultures. **a** Flow cytometry results from cells collected after 4 weeks of NBM cultures where data are plotted as means ± SEM (*n* *=* *3* for the 2D MNC cultures and *n* *=* *6* for 3D MNC cultures and 3D CD34^+^ cultures). For 2D MNC vs. 3D CD34^+^ cultures *p* = 0.0006 for non-erythroid hematopoietic cells, *p* = 0.0008 for erythroid progenitors and *p* = 0.0087 for intermediate erythroblasts. For 3D MNCs vs. 3D CD34^+^ cultures *p* = 0.0014 for non-erythroid hematopoietic cells, *p* = 0.0006 for erythroid progenitors and *p* = 0.0169 for intermediate erythroblasts. No viable cells were detected for the CD34^+^ 2D cultures. **b** Flow cytometry results from cells collected after 4 weeks of MDS-RS cultures where data are plotted as means ± SEM (*n* *=* *5* for the 2D MNC, *n* *=* *6* for the 3D MNC cultures and *n* *=* *7* 3D CD34^+^ cultures). For 3D MNC vs. 3D CD34^+^ cultures *p* = 0.03 for intermediate erythroblasts. No viable cells were detected for the CD34^+^ 2D cultures. One-way ANOVA followed by Tukey’s HSD post hoc test was used for all calculations. **c** May-Grünwald Giemsa stained cells from 4 weeks of NBM cultures in indicated conditions, where erythroid progenitor cells are marked with (*) asterisk, intermediate erythroblasts with ‘>’ symbol, and enucleated erythrocytes with ‘→’ symbol; scale bar, 100 μm. **d** May-Grünwald Giemsa stained cells from 4 weeks of MDS-RS cultures in indicated conditions, where erythroid progenitor cells are marked with (*) asterisk, intermediate erythroblasts with ‘>’ symbol, and enucleated erythrocytes with ‘→’ symbol; scale bar, 100 μm

To assess the requirement of mature hematopoietic and non-hematopoietic BM cells to support erythroid cultures, BM MNCs or CD34^+^ cells were seeded into scaffolds (3D) and suspension (2D) cultures. The cultures were maintained for up to four weeks with identical medium composition followed either by cell extraction or whole scaffold fixation, embedding and sectioning for readout experiments (Fig. 1a). The CD34^+^ 2D cultures did not contain any viable cells at the end of the 4 week period, therefore results from the second week of culture, when a sufficient amount of viable cells for experimentation could still be collected, are depicted in Supplementary Fig. 2. To investigate the capacity of the different culture models to support erythroid differentiation, cells were harvested after 4 weeks of culture and analyzed by flow cytometry for presence of non-erythroid hematopoietic cells (Aqua^−^CD45^+^CD235a^−^), erythroid progenitor cells (Aqua^−^CD45^−^Draq5^+^CD235a^medium^), intermediate erythroblasts (Aqua^−^CD45^−^Draq5^+^CD235a^high^) and enucleated erythrocytes (Aqua^−^CD45^−^Draq5^−^CD235a^+^) [32, 33] (Fig. 1b, c and Supplementary Fig. 3). Harvested cells were also fixed and stained with May-Grünwald Giemsa for morphological visualization (Fig. 2c, d and Supplementary Fig. 2b, c).

When culturing NBM MNCs, the 2D cultures predominantly facilitated growth of non-erythroid hematopoietic cells (91.2 ± 3.4%) with only 7.2% (±3.6) total erythroid cells detected after 4 weeks of culture (Fig. 2a, c). The MNCs cultured in 3D scaffolds generated a higher proportion of total erythroid cells (24.1% ± 11.3), including 4.5% (±3.1) enucleated erythrocytes. Importantly, albeit not surviving past 3 weeks in 2D, the CD34^+^ cells cultured in 3D expanded for 4 weeks and generated the highest percentage of total erythroid cells, 62.8% (±4.8) with a significantly higher proportion of erythroid progenitors and intermediate erythroblasts compared to both MNC cultures (Fig. 2a, c). Considering the fact that medium supplementation was identical with the 2D culture system, these data strongly suggest that the 3D structure allows for organization, growth and maturation of healthy erythropoiesis that cannot be provided by the 2D system.

To test if the cultures could recapitulate aberrant erythropoiesis we cultured BM cells from eleven *SF3B1* mutated MDS-RS patients. We chose MDS-RS patients with stable hemoglobin levels, suggesting that their BM still contained progenitor cells capable of endogenous erythrocyte production (Supplementary Table 1). MDS-RS erythropoiesis was favored in the 3D CD34^+^ cultures, with 33.4% (±6.9) total erythroid cells and 4.5% (±2.2) enucleated erythrocytes after 4 weeks of culture (Fig. 2b, d). By contrast the 2D and 3D MNC cultures generated a lower proportion of erythroid cells (17.6 ± 8.7 and 14.1% ± 6.1, respectively), and very few mature erythrocytes (0.8 ± 0.3 and 0.8 ± 0.5%, respectively) (Fig. 2b, d). The MDS-RS 3D CD34^+^ cultures generated a significantly higher proportion of intermediate erythroblasts compared to the corresponding 3D MNC cultures. Comparing MDS-RS 3D CD34^+^ and MNC cultures, the former generated a higher proportion of intermediate erythroblasts (*p* = 0.03 for 3D MNC and *p* = 0.055 for 2D MNC cultures). We have previously shown that the intermediate erythroblasts include the ring sideroblasts [17].

MDS-RS CD34^+^ 3D culture generated a significantly lower percentage of erythroid progenitors and intermediate erythroblasts compared to NBM cultures (Supplementary Fig. 4c), which is to be expected considering the well-described lower potential for erythroid differentiation in this disease [17]. No significant differences were observed between erythropoiesis in NBM and MDS-RS MNC cultures (Supplementary Fig. 4a, b).

### Proliferation and in vitro generation of erythroblastic islands

**Fig. 3:**
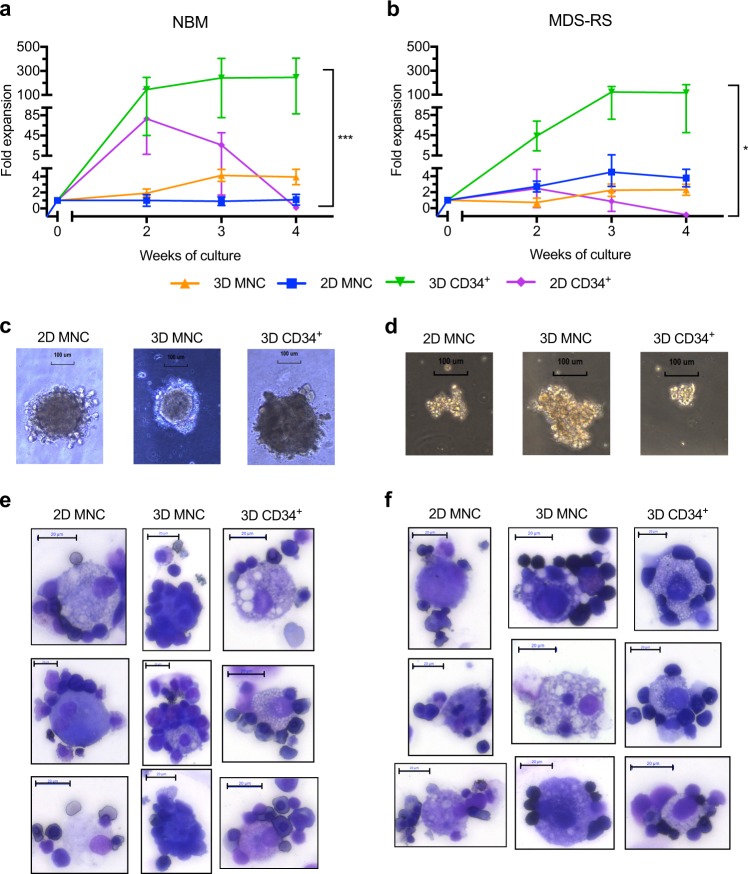
Cell expansion, long term maintenance of progenitor cells and formation of erythroblastic islands. **a** Cell proliferation measured using the MTS assay of NBM cultures over a 4-week period (*n* *=* *3)*. *p* = 0.0003 for 2D MNCs vs. 3D CD34^+^ culture, *p* = 0.0004 for 3D MNCs vs. 3D CD34^+^ culture and *p* = 0.0011 for 3D CD34^+^ vs. 2D CD34^+^ culture. **b** Cell proliferation measured using the MTS assay of MDS-RS cultures over a 4-week period (*n* *=* *5* for 2D MNC*, n* *=* *4* for 3D MNC, *n* = 3 for 3D CD34^+^ and *n* *=* *6* for 2D CD34^+^ culture). *p* = 0.0299 for 2D MNCs vs. 3D CD34^+^ culture, *p* = 0.0265 for 3D MNCs vs. 3D CD34^+^ culture and *p* = 0.0250 for 3D CD34^+^ vs. 2D CD34^+^ culture. All data are plotted as mean (±SEM) expansion after normalization with control medium/scaffolds. One-way ANOVA followed by Tukey’s HSD post hoc test was used for calculations. **c** Long-term culture-colony forming cell (LTC-CFC) colonies derived from cells extracted after week 4 of NBM culture in indicated conditions; scale bar, 100 μm. Experiments were repeated two times. **d** LTC-CFC colonies derived from cells extracted after week 4 of MDS-RS cultures in indicated conditions; scale bar, 100 μm. Experiments were repeated two times. **e** Three representative images of erythroblastic islands from week 4 of 3D MNC, 2D MNC, and 3D CD34^+^ cultures of NBM stained with May-Grünwald Giemsa; scale bar 20 μm. **f** Three representative images of erythroblastic islands from week 4 of 3D MNC, 2D MNC, and 3D CD34^+^ cultures of MDS-RS samples stained with May-Grünwald Giemsa; scale bar 20 μm

The *in situ* proliferation assay MTS [34] was used to determine cell proliferation as a measure of metabolic activity, and was calculated as the relative expansion compared to input cells at the day of seeding (starting value = 1). MNCs from NBM maintained stable cell proliferation (1.09 ± 0.68) throughout four weeks of 2D cultures while they expanded four fold (3.94 ± 0.96) in 3D culture (Fig. 3a). Interestingly, while CD34^+^ cells in 2D culture lost proliferative capacity after 2 weeks of culture, the CD34^+^ cells expanded the most of all cultured cells in 3D (246.95 ± 159.00) (Fig. 3a).

MDS-RS MNCs expanded four fold (3.78 ± 1.10) in 2D cultures (Fig. 3b) compared to two fold in 3D (2.31 ± 0.70) (Fig. 3b). Similar to the NBM cultures, CD34^+^ cells from MDS-RS patients expanded most of all cultured cells in 3D (117.18 ± 66.79) while they stopped proliferating after 2 weeks in 2D culture (Fig. 3b). These results suggest that the CD34^+^ cells have a proliferative advantage when cultured in 3D compared to 2D, both for healthy and dysregulated erythropoiesis, and that CD34^+^ cells have a higher proliferative potential than MNCs, which is to be expected since the MNCs include mature cells.

To assess if cells with self-renewal capacity were retained in the cultures, cells extracted after 4 weeks of culture were plated into long-term culture colony-forming cell (LTC-CFC) assays. Cells obtained from 2D MNC, 3D MNC, and 3D CD34^+^ cultures gave rise to LTC-CFCs both for NBM and MDS-RS (Fig. 3c, d), while no cells could be investigated from week 4 of 2D CD34^+^ cultures. As we previously showed that LTC-CFC growth is highly variable between MDS-RS samples [17] and the method is not developed to assess activity of MNCs or after long-term culture, we only detected positive or negative growth and did not compare NBM and MDS-RS LTC-CFC growth in this system. Nevertheless, considering that the cells preserved the ability to sustain long-term culture resulting in detection of colonies, these data suggests that the cultures retain stem and progenitor cells.

Generation of functional terminal erythropoiesis includes formation of erythroblastic islands, which would be an essential component of an erythroid culture model [3–5]. We observed generation of erythroblastic islands in the 2D MNC, 3D MNC, and 3D CD34^+^ cultures of NBM and MDS-RS samples (Fig. 3e, f). We evaluated the size of erythroblastic islands by counting the number of erythroid cells attached to each macrophage but did not observe significant differences between the NBM and MDS-RS cultures (Supplementary Fig. 5a), in analogy with a previous report of morphological assessment of primary MDS-RS BM smears [35]. To visualize proliferation and differentiation of MNCs and CD34^+^ cells within the 3D cultures, scaffolds were fixed and paraffin embedded after 4 weeks of culture followed by sectioning and staining. We found that both the MNC and CD34^+^ 3D cultures supported the formation of large clusters of erythroid cells (Supplementary Fig. 5b, c). Finally, we also visualized erythroblastic islands *in situ* using fluorescent staining and confocal microscopy of fixed scaffolds (Supplementary Video 1).

### Cytokine secretion in 3D cultures

To evaluate if factors known to have an effect on erythropoiesis and to be altered in MDS-RS [36–38] were differentially secreted in NBM and MDS-RS cultures we measured the concentration of transforming growth factor (TGF)-β1, growth differentiation factor 11 (GDF11), interleukin 10 (IL-10) and interleukin 1 alpha (IL-1α) in medium collected from MNC and CD34^+^ 3D cultures at week 1, 2, and 4.

**Fig. 4:**
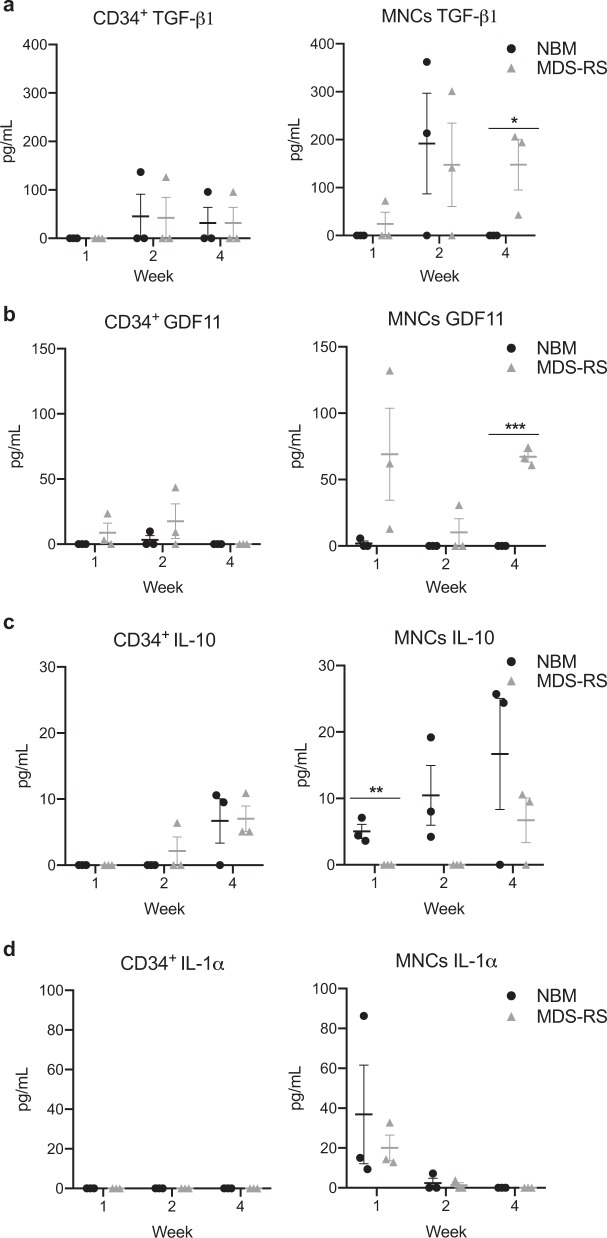
Secretion of TGF-β1, GDF11, IL-10, and IL-1α during four weeks of NBM and MDS-RS 3D cultures. **a** Concentration of TGF-β1 in pg/ml measured at the end of week 1, 2, and 4 of 3D CD34^+^ (left) and MNC (right) 3D cultures (*n* *=* *3)*, with *p* = 0.048 for week 4 of MNC cultures. **b** Concentration of GDF11 in pg/ml measured at the end of week 1, 2, and 4 of 3D CD34^+^ (left) and MNC (right) 3D cultures (*n* *=* *3)*, with *p* < 0.001 for week 4 of MNC cultures. **c** Concentration of IL-10 in pg/ml measured at the end of week 1, 2, and 4 of 3D CD34^+^ (left) and MNC (right) 3D cultures (*n* *=* *3)*, with *p* = 0,001 for week one of MNC cultures. **d** Concentration of IL-1α in pg/ml measured at the end of week 1, 2, and 4 of 3D CD34^+^ (left) and MNC (right) 3D cultures (*n* *=* *3)*. The data is plotted as means ± SEM and an unpaired two-tailed *t*-tests was used for calculations

No significant differences between MDS-RS and NBM were observed in the CD34^+^ 3D cultures, which overall produced low cytokine levels (Fig. 4). TGF-β1, known to block proliferation of erythroid cells [39–41], was detected during the second week of NBM MNC cultures but then disappeared at four weeks (Fig. 4a), reflecting effective erythroid expansion (Fig. 3a). By contrast, TGF-β1 concentration was stable between week two and four of MDS-RS cultures, characterized by ineffective erythroid output (Fig. 3b). Hence TGF-β1 secretion was higher in MDS-RS than in NBM cultures, in line with reports of sustained TGF-β signal activation in MDS [37, 38].

While GDF11 was not detected in NBM cultures secretion was increased in MDS-RS (Fig. 4b). This is in accordance with reports showing that GDF11 concentration is higher in serum of MDS patients compared to healthy individuals [36, 42]. In addition, we found that IL-10, which synergizes with Epo to increase erythroid differentiation [43], was increased throughout cultures (Fig. 4c) and IL-1α, an inhibitor of erythroid maturation [44], was lost from the second week (Fig. 4d), reflecting the erythroid maturation observed in the cultures (Fig. 2).

### In vitro maintenance of the *SF3B1* mutated clone and de novo generation of ring sideroblasts

**Fig. 5:**
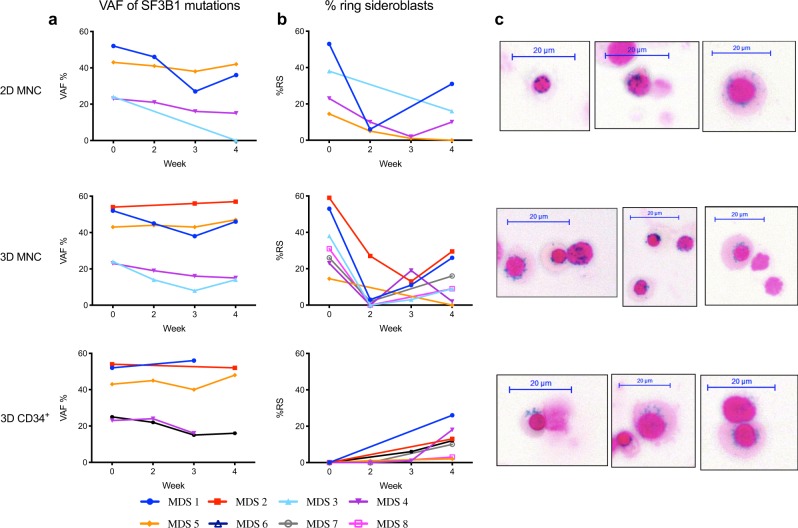
Maintenance of the MDS-RS clone and de novo ring sideroblast generation. **a** Variant allele frequency (VAF%) of *SF3B1* mutations measured throughout 4 weeks of indicated MDS-RS cultures using pyrosequencing. **b** Percentage of ring sideroblasts (RS) as visualized by Perl´s Prussian blue staining throughout 4 weeks of indicated MDS-RS cultures. **c** Representative cytospin images of RS stained with Prussian blue generated after 4 weeks of indicated cultures; scale bar, 20 μm

To allow for in vitro assessment of clonal erythropoiesis it is essential that the culture model maintains the mutated clone until late erythropoiesis, as observed in the BM of patients with MDS-RS [11, 27]. Here we used pyrosequencing to detect the *SF3B1* mutant allele during the 4 week culture period. Variant allele frequency (VAF) of *SF3B1* was maintained for all individual samples tested in the 3D cultures, and equally well for scaffolds seeded with MNCs and CD34^+^ cells (Fig. 5a). Mutant *SF3B1* VAF was maintained for 3 out of 4 patients in the 2D MNC cultures (Fig. 5a), and for the first 2 weeks analyzed in 2D CD34^+^ cultures (Supplementary Fig. 2d).

Cultures were additionally analyzed for formation of RS, using the same methodology as in routine morphological analysis of BM smears. MNCs obtained from patients with MDS-RS contained RS at seeding, corresponding to their frequency in the BM, but after two weeks in the MNC cultures, most of these seeded RS had disappeared (Fig. 5b; Supplementary Table 1). As previously observed 2D CD34^+^ cultures failed to generate RS (Supplementary Fig. 2) [15, 16]. Importantly, however, we detected RS at week 4 in all individual CD34^+^ 3D, MNC 2D and 3D cultures, with evidence of de novo generation in the majority of cultures (Fig. 5b, c). This is the first time that effective de novo generation of RS from MDS-RS primary cells has been reported in vitro.

## Discussion

Ineffective erythropoiesis, severe anemia, and chronic transfusion need are hallmarks of a number of benign and malignant conditions, including hemoglobinopathies and myelodysplastic syndromes [10, 45]. One example is the markedly hyperplastic erythropoiesis and terminal erythroid maturation defect associated with *SF3B1*-mutated MDS-RS [8, 11]. Our group has previously shown that the iron transporter ABCB7 mediates the phenotype of MDS-RS, but the paucity of experimental models that reliably recapitulate RS formation limits further studies of this pathway [27]. In addition, this lack of ex vivo models restricts the possibility for effective screening of novel therapeutic compounds with a potential to target perturbed terminal erythropoiesis, such as luspatercept and sotatercept, where the mechanisms of action has been assessed in mouse models [42, 46], while their effects in human erythropoiesis are yet to be elucidated.

Here, we describe an in vitro culture system able to support terminal human erythropoiesis including formation of erythroblastic islands and enucleated red cells. Importantly, the system supports erythropoiesis from patients with *SF3B1* mutated MDS-RS. For the first time, de novo production of RS from MDS-RS primary cells is supported by an in vitro culture system. Moreover, the mutated *SF3B1* variant allele burden remains stable throughout the maturation process, in analogy with what we have demonstrated in primary MDS-RS aspirates [11].

Based on previous reports that the addition of autologous mesenchymal BM cells facilitated the engraftment of human MDS hemopoiesis in NSG mice [47] we set out to test the hypothesis that the addition of other autologous mononuclear BM cells than CD34^+^ would facilitate proliferation, as well as erythroid maturation in the cultures. Indeed, the addition of the MNC component to the suspension cultures supported cell survival and proliferation beyond two weeks and facilitated formation of erythroblastic islands and RS. By contrast, CD34^+^ 2D cultures did not proliferate beyond 2 weeks and generated no RS. Hence, the MNC component did contribute to proliferation and erythroid maturation in the 2D and 3D cultures.

Interestingly, the 3D scaffold system supported long term expansion and erythroid proliferation and maturation of CD34^+^ cells and gave rise to a median of 62,8% (±4.845) of erythroid cells, a significantly higher percentage than both the 2D MNC (*p* = 0004) and 3D MNC (*p* = 0013) cultures. This is in accordance with previous publications showing that co-culturing CD34^+^ cells with mesenchymal stromal cells [48], or with conditioned media produced by BM stromal cells [49], has a negative effect on erythropoiesis in favor of the myeloid lineage. In addition, the drastic difference between proliferative and survival capacity of CD34^+^ cells in 2D and 3D might stem from the capability of the cells to form cellular niches in the 3D environment, giving rise to concentration gradients of secreted factors [48, 50] representative of what occurs in vivo.

It has recently been shown that xenotransplantation of MDS-RS HSCs give rise to erythroid maturation and RS formation in immunocompromised mice [17, 18], implicating the importance of the 3D BM microenvironment to support terminal differentiation of aberrant MDS-RS erythropoiesis. We therefore utilized scaffolds previously shown to maintain in vitro culture of acute myeloid leukemia cell lines [23], healthy cord blood MNCs [24] and peripheral blood CD34^+^ cells [51] making them ideal to provide spatial niches resembling the BM architecture and hopefully allowing for cell-cell and cell-matrix interactions. Indeed the 3D scaffolds provided a microenvironment that supported the formation of erythroblastic islands while also maintaining LTC-CFC generating capacity after 4 weeks of culture. While the scaffolds supported both MNC and CD34^+^ growth, the latter proved to be more effective in terms of cell expansion and support of erythropoiesis. The present study therefore supports the importance of the 3D structure rather than the presence of autologous stromal cells to facilitate erythropoiesis, albeit a synergy between these two cannot be excluded.

The human BM 3D culture system constitutes a novel, effective and easy way to model human erythropoiesis, including MDS-RS where erythroid failure is associated with the acquisition of somatic mutations. The CD34^+^ system is more effective with regard to erythroid output and makes it easier to control for cellular input and recovery, and is effective using thawed vital-frozen BM cells, which is critical for using existing biobanked material. In addition, since RS is not a part of the CD34^+^ cell fraction seeded into the scaffolds, but is generated de novo throughout culture, this may be a preferable model to study RS formation. Although the 3D MNC culture did not facilitate erythropoiesis or cellular expansion to the same degree as the CD34^+^ 3D culture, they did facilitate the secretion of factors known to have an effect on erythropoiesis to a higher extent than the CD34^+^ 3D cultures. Therefore, we suggest that this system could be of interest when studying the effects of stroma or other non-erythroid hematopoietic cells on erythropoiesis in the BM. The model we present offers the exciting possibility to dissect physical, molecular, and cellular components critical for terminal erythropoiesis and opens a new door to the implementation of high-throughput screening of pharmaceutical compounds for erythroid failure disorders by offering an easier and cheaper alternative to the use of animal models.

## Supplementary information

Supplementary video 1

Supplements
