# Supplementary material for: A three-dimensional in vitro model of erythropoiesis recapitulates erythroid failure in myelodysplastic syndromes
Source: Leukemia. 2019 Aug 2;34(1):271–82. doi: 10.1038/s41375-019-0532-7 (PMC7214248; doi:10.1038/s41375-019-0532-7)
Supplement: Supplementary file 2 — Supplements [file 41375_2019_532_MOESM2_ESM.docx]

**Supplementary methods**

*Culture conditions*

In order to promote erythroid differentiation, three different medium compositions were sequentially used to support the culture. During the first week, cells were cultured in Iscove**’**s Modified Dulbecco**’**s Medium (IMDM; Sigma-Aldrich, St. Louis, Missouri, USA) with GlutaMAX™ supplement (Invitrogen, Waltham, Massachusetts, USA), 1% Penicillin/Streptomycin (Invitrogen), 15% BIT9500 serum substitute (StemCell Technologies, Vancouver, British Columbia, Canada), 25 ng/ml recombinant human stem cell factor (hSCF; Invitrogen), 10 ng/ml recombinant human interleukin 3 (IL-3, Invitrogen) and 10 ng/ml recombinant human interleukin 6 (IL-6; Invitrogen). From the second week, erythropoietin (Epo; PBL Assay Science, Piscataway, New Jersey, USA) at a concentration of 2 IU/ml (units per ml) and iron saturated human holo-Transferrin (Sigma-Aldrich) at a concentration of 800 ng/ml were added to the medium. From the third week BIT9500, IL-3 and IL-6 were removed from medium and 10% fetal bovine serum (FBS; Invitrogen) was added during the last two weeks of culture. In the 3D cultures the medium was changed every 2-3 days by aspiration and replaced with 1.5 ml fresh media. In the 2D cultures, to avoid losing cells, the medium was first gently aspirated to avoid removing the bulk of the cells, and then spun down to collect any aspirated cells that were resuspended in 1.5 ml of fresh medium, which was later added back to the corresponding 2D MNCs and the CD34^+^ cell cultures. Aspirated cells were counted using a standard hemocytometer and trypan blue dye before they were divided between different assays.

*Scaffold fabrication and sterilization*

Each scaffold was cut into cubes of approximately 0.5 cm^3^, coated with collagen type I from calf skin (Sigma-Aldrich) at a concentration of 62.5 μg/ml as previously described^23^, sterilized by exposure to UV light (v230, 50 Hz, 0.8AMP, Herolab, Germany) for 10 minutes followed by gamma irradiation (2.5 mRAD) and finally immersion in Ethanol (70% v/v) for 2 hours (Supplementary Figure 1). The scaffolds were then washed twice for 10 minutes in Phosphate Buffered Saline (PBS, Thermo Fisher Scientific, Waltham, Massachusetts, USA), before adding medium (IMDM + 1% Penicillin/Streptomycin) and placing them in a humidified incubator for 7 days at 37°C and 5% CO_2_ prior to use.

*Flow cytometry*

MNCs from day 0 and cells extracted at week 2 and 4 of cultures were stained with the following antibodies; FITC conjugated anti-human CD45 (BD Biosciences, #555482, clone HI30), Draq5 to identify nucleated cells, e450 conjugated anti-human CD235a and LIVE/DEAD® Fixable Aqua Dead Cell Stain (Thermo Fisher Scientific, #L34965) to exclude dead cells. The cells were treated with FcR blocking reagent (Miltenyi Biotec) for 15 minutes at room temperature before adding Aqua for 45 minutes at 4°C in the dark. Aqua was washed off and the cells were stained with antibodies for 45 minutes at 4°C in the dark. Single stain controls were used for compensation and fluorescent minus one (FMO) controls^32^ were included where cell numbers allowed (Supplementary Table 3). If FMOs could not be included due to low cell number, the results from FMOs from the closest time-point of the same experiment were used for analysis. Samples were run on an LSRFortessa™ instrument (BD Biosciences), equipped with 405 nm, 640 nm, 488 nm and 561 nm lasers, at the Karolinska Institute MedH flow cytometry facility. Data analysis was performed using the FlowJo V.10 analysis soft­ware (Tree Star) and gating strategy (Figure 1a,b and Supplementary Figure 3) was based on a combination of biologically separate populations with support from the FMO controls. The populations were plotted as mean percentage (with standard error of the mean; SEM) of total viable hematopoietic cells (where CD45^-^GPA^-^ cells were excluded). This was done to avoid bias between the 2D and 3D cultures in regards of absolute numbers since all cells could be not be extracted from the scaffolds.

*Cell proliferation*

During the first week of culture we aimed to avoid stressing the cells and let them settle by not including factors that drive erythropoiesis in the medium. For that reason and to save cell numbers for other readouts, we did not include that time point in the proliferation measurements. For the 3D cultures and 2D MNC cultures, 200 μl of the MTS solution was added directly to wells containing 1 ml of medium, and for the 2D CD34^+^ culture 20 μl of the tetrazolium compound were added to 100 μL of cell suspension. These were incubated for 4 hours at 37°C and 5% CO_2_. After incubation absorbance was measured at 492 nm using a 96-well plate-reader (Infinite 200 PRO multimode reader, Tecan, Switzerland) and calculated according to an internal standard. For the 3D cultures, empty scaffolds immersed in medium were used as blanks and for the liquid cultures medium only was used as blank. Results were graphed as mean fold expansion (with SEM) related to the day of seeding (starting value = 1).

*Functional stem and progenitor cell assays*

Functional long-term culture colony-forming cell (LTC-CFC) assays were performed as previously described^32, 33^. Briefly, cells were extracted after 4 weeks of culture and 250 cells from CD34^+^ cultures or 0.02 - 0.1 x 10^6^ cells from MNC cultures were plated in triplicates onto irradiated (80 Gy) murine stromal feeders (M2-10B4 and SI/SL) on collagen-coated 96 well (CD34^+^ cultures) or 24 well (MNC cultures) plates in the presence of MyeloCult H5100 medium (Stem Cell Technologies) with 10^-6^M hydrocortisone 21-hemisuccinate (Stem Cell Technologies). After 6 weeks of culture at 37°C and 5% CO_2_ with weekly half-media exchange, cells were harvested and plated into MethoCult H4434 media (Stem Cell Technologies). Colony forming cells were scored under an inverted microscope after 14 days (Leica DM microscope; Leica Microsystems, Wetzlar, Germany).

*Pyrosequencing*

DNA from cells aspirated at day 0 and at the end of weeks 2, 3 and 4 from the MDS-RS cultures was extracted (GenElute^™^ Mammalian Genomic DNA Miniprep Kit, Sigma-Aldrich). DNA from healthy BM was used as wild type controls and DNA from CD3^+^ T-cells (selected via MACS (Miltenyi Biotech)) from the MDS-RS patients were used as internal controls. PCR amplification was performed according to manufacturer’s protocol. PCR was run at 95°C for 10 minutes, 40 cycles of 95°C for 15 seconds, 56°C for 30 seconds and 72°C for 45 seconds. Reaction mixtures for pyrosequencing were prepared in 25 μl volume of: SYBR Green mix (Applied Biosystems, Foster City, CA, USA), 0.4mM sequencing primer, 10 ng/μl of DNA and nuclease-free water (Life Technologies Corporation, Carlsbad, CA, USA). Mutation-specific assays were designed via PyroMark assay design software (Qiagen, Hilden, Germany) and performed according to the PyroMark Q24 user manual. The limit of detection of all the assays included was validated with healthy control DNA and set at 5% (data not shown).

*Measurements of secreted factors*

TGF-β1 was measured with a bead-based multiplex assay using the Luminex^®^ technology (MILLIPLEX^®^ MAP TGFß Magnetic Bead 3 Plex Kit - Immunology Multiplex Assay, Merck KGaA, Darmstadt, Germany) according to manufacturer´s instructions with a minimum detectable concentration of 6.0 pg/ml based on manufacturer specifications. Growth differentiation factor 11 (GDF11) was measured using a sandwich enzyme-linked immune-sorbent assay (Human GDF11 (Growth/differentiation factor 11) ELISA kit, Nordic Biosite, Täby, Sweden) according to manufacturer’s protocol, where absorbance was measured at 450nm using a 96-well plate-reader (Infinite 200 PRO multimode reader, Tecan) with a range of detectable concentration of 15.635 – 1000pg/ml, according to manufacturer. IL-10 and IL-1α were measured with a bead-based multiplex assay using the Luminex^®^ technology (MILLIPLEX^®^ MAP Human Cytokine/Chemokine Magnetic Bead Panel - Immunology Multiplex Assay, Merck KGaA) according to manufacturer´s instructions with a minimum detectable concentration of 1.1 pg/ml for IL-10 and 9.4 for IL-1α based on manufacturer specifications. For all measurements standard curves of known concentrations of recombinant human cytokines were used to convert fluorescent units or absorbance levels to cytokine concentration units (pg/ml) and supernatants from empty scaffolds treated identically to scaffolds containing cells were used as blanks.

*Morphological and histopathological evaluation*

Cells were spun onto microscope slides (0.025 x 10^6^ cells per slide) followed by fixation in methanol for 8 minutes and stained with May-Grünwald (10% in phosphate buffer, pH 6.8) - Giemsa (50% in phosphate buffer, pH 6.8) for morphological evaluation and counting erythroid progenitors surrounding macrophages in erythroblastic islands. The paraffin embedded scaffolds were sectioned (25 μm) and stained with hematoxylin and eosin (H&E). The frozen scaffolds were sectioned (25 μm), washed and stained overnight at 4°C with e450 conjugated anti-human CD235a (eBioscience, #48-9884-42, clone 6A7M), FITC conjugated anti-human CD68 (Biolegend, #333806, clone Y1/82A) and Draq5 nuclear stain (eBioscience, #65-0880-92).

**Supplementary table 1. Patient characteristics.**

| **Patient** | **Treatment*** | **BM** | **WBC** | **ANC** | **Plt** | **Hb** | **Ery**  **(%)** | **RS**  **(%)** | **Blasts** | **TD** | ***SF3B1 Mutation*** | ***SF3B1* VAF** |
| --- | --- | --- | --- | --- | --- | --- | --- | --- | --- | --- | --- | --- |
|  |  | **Cellularity (%)** | **(10^9^/l)** | **(10^9^/l)** | **(10^9^/l)** | **(g/dl)** |  |  | **(%)** |  |  |  |
| **MDS 1** | Epo | 60 | 6,1 | 2,9 | 266 | 9,5 | 45 | 53 | 0 | No | K700E | 0,52 |
| **MDS 2** | Epo | 60 | 5,3 | 3,3 | 307 | 10,2 | 33 | 59 | 1 | No | K666R | 0,54 |
| **MDS 3** | Epo | 50 | 5,3 | 3 | 304 | 10,3 | 28 | 38 | 2 | No | E622D | 0,24 |
| **MDS 4** | None | 30 | 6,7 | 3,8 | 279 | 12,0 | 30 | 23 | 1,2 | No | N626D | 0,23 |
| **MDS 5** | Epo | 60 | 3,8 | 1,3 | 475 | 9,9 | 46 | 14,5 | 2,4 | No | H662Q | 0,43 |
| **MDS 6** | None | 70 | 5,6 | 3,2 | 348 | 10,6 | 26 | 17,5 | 3 | No | H662Q | 0,25 |
| **MDS 7** | Epo | 60 | 6,4 | 3,4 | 372 | 100 | 40 | 39 | 1,5 | No | M784_K785delinsI | 0,38 |
| **MDS 8** | None | 50 | 7 | 4,3 | 261 | 115 | 27 | 38 | 1 | No | K700E | 0,35 |
| **MDS 9** | None | 40 | 4.3 | 1,6 | 343 | 113 | 45 | 62 | 0 | No | K700E | 0,35 |
| **MDS 10** | None | 40 | 4,5 | 2,1 | 341 | 11,0 | 30 | 40 | 2,5 | No | K700E | 0,41 |
| **MDS 11** | None | 50 | 7 | 4,7 | 316 | 10,2 | 35 | 66 | 1,5 | No | K700E | 0,37 |

BM Cellularity (%) = bone marrow cellularity (percent), WBC = white blood cells (10^9^/l), ANC = absolute neutrophil count (10^9^/l), Plt = platelets (10^9^/l), Hb = hemoglobin (g/dl), Ery (%) = percent erythropoietic cells of all nucleated bone marrow cells, RS % = percent ring sideroblasts of all nucleated erythroid bone marrow cells, *SF3B1* VAF = Variant allelic frequency as identified by pyrosequencing of bone marrow MNCs^14, 32^. Epo = erythropoietin *

**Supplementary Table 2. Number of CD34^+^ cells seeded per scaffold.**

| **Sample** | **MNCs** | **CD34^+^ cells** | **CD34^+^ / scaffold** | **% CD34^+^** |
| --- | --- | --- | --- | --- |
| NBM 2 | 29210000 | 779000 | 53338 | 2,67 |
| NBM 3 | 63210000 | 741000 | 23446 | 1,17 |
| NBM 4 | 40000000 | 1060000 | 53000 | 2,65 |
| NBM 7* | 50000000 |  |  |  |
| NBM 8* | 50000000 |  |  |  |
| NBM 9* | 50000000 |  |  |  |
| MDS 1 | 40000000 | 1080000 | 54000 | 2,70 |
| MDS 2 | 70000000 | 2014000 | 57543 | 2,88 |
| MDS 4 | 134000000 | 2680000 | 40000 | 2,00 |
| MDS 8 | 250000000 | 4104000 | 32832 | 1,64 |
| MDS 6 | 112494978 | 4200000 | 74670 | 3,73 |
| MDS 7 | 77000000 | 1320000 | 34286 | 1,71 |
| MDS 8 | 115000000 | 960000 | 16696 | 0,83 |
| MDS 9 | 63000000 | 990000 | 31429 | 1,57 |

MNCs = MNCs used for CD34^+^ enrichment, CD34^+^ cells = number of cells after enrichment, % CD34^+^ = percentage of CD34^+^ cells in MNC fraction, CD34^+^/scaffold = number of CD34^+^ cells seeded into each scaffold, *Equivalent numbers of MNCs from the same patient were seeded into scaffolds and used for CD34^+^ enrichment followed by seeding into the same number of scaffolds that received the corresponding MNCs, CD34^+^ cells were not counted after enrichment

**Supplementary table 3. FMO inclusion in each experiment.**

| **Sample** | **Week 2** | **Week 4** |
| --- | --- | --- |
| NBM 1 | x | Aqua |
| NBM 2 | x | x |
| NBM 3 | x | x |
| NBM 4 |  | x |
| NBM 7 |  | x |
| NBM 8 |  | x |
| NBM 9 |  | x |
| MDS 1 | x | x |
| MDS 2 | x | x |
| MDS 3 |  | x |
| MDS 4 |  | x |
| MDS 5 |  | 0 |
| MDS 6 |  | 0 |
| MDS 7 |  | x |
| MDS 9 |  | x |
| MDS 10 | x |  |
| MDS 11 |  | x |

x= FMOs were included, 0= FMOs were not included and therefore FMOs recorded from week 3 of culture were used, Aqua= only FMO for Aqua recorded

**Supplementary table 4. Primers for pyrosequencing.**

| **Assay ID** | **Fwd Primer** | **Rev Primer** | **Sequencing** | **Sequence to analyze** |
| --- | --- | --- | --- | --- |
| **SF3B1_H625** | TTAGGCTGCTGGTCTGGCTACTAT | GAGGCTACAACAGCAAAAGCTCTA | ATAACATGGATGAGTATGTC | YKTAACACAA CAGCTAGAGC TTTTGC |
| **SF3B1_H662** | CATGGATGAGTATGTCCGTAACAC | TTCTAAGATGTGGCAAGATGGC | AAGTCCTGGCAAGCG | AGASAVACTG GTATTAAGAT TGTACAACA |
| **SF3B1_H666** | CATGGATGAGTATGTCCGTAACAC | TTCTAAGATGTGGCAAGATGGC | AAGTCCTGGCAAGCG | AGACACACTG GTATTRRBAT TGTACAACAG ATAGCTATTC T |
| **SF3B1_K700E** | GTGTTTGGTTTTGTAGGTCTTGTG | TACCATAAGGAGTTGCTGCTTCAG | ACTGATGGTCCGAACT | TYCTGCTGCT CATCCACAAG ACCTACA |

All the above primers and probes were ordered from biomeres.net (Ulm, Germany)

**Supplementary Figure 1.** Scaffold fabrication and sterilization. (**a**) Polyurethane solution was made by melting pellets at 50°C with a chemical solvent and then transferred to a petri dish and frozen at -80°C for two hours, (**b**) Thermally induced phase separation was used to separate out the solvent, (**c**) resulting in a polyurethane sponge. (**d**) The sponge was cut into scaffolds of approximately 0.5 cm^3^ and (**e**) coated with collagen type I before (**f**) sterilization with UV irradiation (v230, 50 Hz, 0.8AMP), gamma irradiation (2.5 mRAD) and Ethanol (70%).

**Supplementary Figure 2.** Results from second week of 2D CD34^+^ cultures**.** (**a**) Cell composition analysed using flow cytometry from cells extracted after 2 weeks of NBM and MDS-RS 2D CD34^+^ culture (*n = 3*) where p=0.0335 for non-erythroid hematopoietic cells and p=0.0051 for intermediate erythroblasts in NBM vs MDS-RS. The data is plotted as means±SEM and two-way ANOVA followed by Sidak´s multiple comparisons test was used for calculations. (**b**) May-Grünwald Giemsa stained cells aspirated from week 2 of NBM culture; scale bar, 100 μm. (**c**) May-Grünwald Giemsa stained cells aspirated from week 2 of MDS-RS culture; scale bar, 100 μm. (**d**) Variant allele frequency (VAF%) of *SF3B1* mutations throughout 2D CD34^+^ culture measured using pyrosequencing. (**e**) Percentage of ring sideroblasts counted using Perl´s Prussian blue staining throughout 2D CD34^+^ culture.

**Supplementary Figure 3**. Flow cytometry strategy**.** Representative flow cytometry analysis for quantifying non-erythroid hematopoietic cells, erythroid progenitor cells (Ery prog), intermediate erythroblasts (Int-EryBs) and enucleated erythrocytes (RBCs) from (**a**) week 4 of NBM 2D MNC culture, (**b**) week 4 of NBM 3D MNC culture, (**c**) week 2 of NBM 2D CD34^+^ culture, (**d**) week 4 of MDS-RS 2D MNC culture, (**e**) week 4 of MDS-RS 3D MNC culture and (**f**) week 2 of MDS-RS 2D CD34^+^ culture.

**Supplementary Figure 4.** Cell composition of NBM vs MDS-RS cells after four weeks of culture. (**a**) Cell composition measured with flow cytometry of MNCs after four weeks of 2D culture with no significant differences found between NBM and MDS-RS samples (NBM *n = 3*; MDS *n= 5*). (**b**) Cell composition measured with flow cytometry of MNCs after four weeks of 3D culture with no significant differences found between NBM and MDS-RS samples (NBM *n = 6*; MDS *n= 6*). (**c**) Cell composition measured with flow cytometry of NBM vs MDS-RS CD34^+^ cells after four weeks of 3D culture (NBM *n = 6*; MDS *n= 7*). The difference was significant for non-erythroid hematopoietic cells (p=0.003), erythroid progenitors (p=0.028), and intermediate erythroblasts (p=0.013). All data are plotted as means±SEM and two-way ANOVA followed by Sidak´s multiple comparisons test was used for calculations.

**Supplementary Figure 5. Size of erythroid islands and erythroid niches inside 3D scaffolds.** (**a**) Number of erythroid cells surrounding each macrophage in erythroblastic islands extracted from week 4 of cultures (*n = 3* for NBM and *n = 4* for all MDS-RS cultures except 2D MNC where *n = 3*). For NBM 3D MNC vs 3D CD34^+^ cultures p*=0.0362. One-way ANOVA followed by Tukey’s HSD post hoc test was used for calculations. (**b**) Hematoxylin-eosin stained scaffold sections from week 4 of 3D MNC and 3D CD34^+^ cultures of NBM; scale bar, 100 μm. (**c**) Hematoxylin-eosin stained scaffold sections from week 4 of 3D MNC and 3D CD34^+^ cultures of MDS-RS; scale bar, 100 μm.

**Supplementary Video 1**. Erythroid island within 3D scaffold. Confocal microscopy video of an erythroid island inside the scaffold after 4 weeks in culture (section 30 μm thick) that had been seeded with MNCs from an MDS-RS sample. Macrophage (CD68) is green, mature erythrocytes

(CD235a) are blue and nuclei (Draq5) are pink. Images were taken at 40X magnification.
